# Quality Characteristics of Baranjski Kulen (PGI) Fermented Sausage from Three Pork Production Chains

**DOI:** 10.3390/foods14203473

**Published:** 2025-10-11

**Authors:** Goran Kušec, Ivona Djurkin Kušec, Kristina Gvozdanović, Miodrag Komlenić, Marina Krvavica, Vladimir Margeta

**Affiliations:** 1 Department of Animal Breeding and Biotechnology, Faculty of Agrobiotechnical Sciences Osijek, Josip Juraj Strossmayer University of Osijek, Vladimira Preloga 1, 31000 Osijek, Croatia; gkusec@fazos.hr (G.K.); kgvozdanovic@fazos.hr (K.G.);; 2 Belje Plus, d.o.o., Svetog Ivana Krstitelja 1a, 31326 Darda, Croatia; 3 Department of Food Technology, The University of Applied Sciences “Marko Marulic” of Knin, Petra Krešimira IV 30, 22300 Knin, Croatia; marina.krvavica@veleknin.hr

**Keywords:** pigs, texture profile, fat content, texture, traditional meat product

## Abstract

The aim of this study was to investigate the physicochemical traits, colour, and texture profile of fermented sausage, Baranjski kulen, produced from the meat of pigs originating from three pork chains. The first pork chain consisted of the Black Slavonian pig breed (PC1), the second pork chain consisted of crossbred pigs from the Croatian breeding programme (Duroc × Large White) (PC2), and the third pork chain (PC3) referred to commercial hybrids (Pietrain × Duroc × Pietrain × Camborough 23). A total of 16 pigs (8 gilts and 8 castrates) from each chain were used, reared to 6–18 months of age, and slaughtered at 135–180 kg. Baranjski kulen from PC2 and PC3 had a higher protein content (up to 2% more) and lower fat content (4–5% less) compared to PC1. PC3 kulen showed greater colour intensity (higher a* values), while PC2 kulen had the highest hardness, cohesiveness, gumminess, and chewiness, indicating a firmer texture. In contrast, PC3 kulen had a softer and more tender texture. These findings underline the impact of production chain on product quality and can be used to optimise processing strategies and strengthen the market potential of Baranjski kulen.

## 1. Introduction

Consumers’ appreciation of local fermented and dried pork products is based on long-standing production tradition, distinctive recipes, and the use of high-quality raw materials, especially meat [1,2]. One of the products that is especially appreciated in Croatia and gained much attention on EU market is Baranjski kulen, a traditional fermented pork sausage from the Baranja region in eastern Croatia, designated with the Protected Geographical Indication (PGI) label. The sausage is made from pig meat, fat, paprika, garlic, a small amount of pepper, and salt. According to the official PGI specification [European Commission, 2014 [3], Baranjski kulen is defined by its firm, oval shape, evenly distributed meat and fat particles, dark red cross-section colour, and a characteristic slightly spicy, smoky flavour with aromas of paprika, garlic, and pepper. These sensory traits are central to its identity and recognition as a traditional product from the Baranja region. The product specification highlights the critical importance of selecting pork as the primary raw material, requiring that the meat for the production of Baranjski kulen is of the highest technological quality [3].

The quality of pork can significantly vary [4] with regard to breed, age, housing systems, feeding strategies, and other premortem factors as well as their interactions [5,6,7]. These factors influence the entire supply chain, from animal characteristics and farm conditions to transport, slaughter, processing, storage, sale, retail, culinary preparation, and consumption [6,8,9].

In the quality assessment of dry/fermented sausages, colour and texture are considered as their two most important attributes [10]. The formation and stability of the colour in dry sausages, hams, and other related dry-cured meat products is closely associated with hydrolytic and oxidative processes in the meat matrix [11]. These involve microbially, enzymatically, and/or chemically catalysed steps, dependent on numerous factors such as pH, pigment concentration, redox potential, curing agent distribution, temperature, and relative humidity. Generally, the characteristic red colour of fermented sausages results from the addition of paprika and the formation of pigment nitrosyl myoglobin (MbFe_2_NO) during the process of curing [11]. It should be noted that the production of dry-fermented sausages on family farms in Croatia, including Baranjski and Slavonski kulen (both with a PGI), is mainly based on drying and fermentation, without the use of nitrites.

The texture of sausage represents another key quality trait that is shaped during fermentation and maturation by enzymatic activity and water reduction. The characteristic texture of dry-fermented sausages is developed through biochemical transformations during fermentation and ripening/drying, driven by proteolytic and lipolytic enzymes. Various factors influence the textural properties of dried fermented sausages, with protein, fat, and moisture content being the most significant. These components interact determining the final texture, each contributing uniquely to the overall sensory experience [12,13].

Protein content is crucial in forming the sausage matrix, particularly through the interaction of myofibrillar proteins and collagen which contribute to the product firmness, elasticity, and shape retention [14]. The amount and type of protein can affect the sausage ability to retain moisture during fermentation and drying, impacting its final texture. Fat, on the other hand, is essential for the juiciness, smoothness, and tenderness of the sausage. Fat droplets disperse within the protein network, acting as a lubricant to prevent the product from becoming too dry or tough. Fat content must be carefully controlled, as excessive fat can lead to a greasy or overly soft texture, while insufficient fat can result in a dry and chewy product. Moisture content, perhaps the most critical factor in determining the texture of dried sausages, directly impacts hardness, chewiness, and overall palatability. As moisture evaporates during the drying process, the sausage becomes firmer and with more pronounced flavour [15].

Based on their peculiarities described by Bonneau and Lebret (2010) [16], the following three alternative chains to produce the raw material for Baranjski kulen are recognised: extended fattening of hybrid pigs on industrial farms; fattening of pigs from the Croatian breeding programme on family farms up to an older age (yearlings); and fattening of the autochthonous breed such as Black Slavonian pig in the traditional way (pasture breeding). By defining these chains, it is possible not only to monitor various qualitative traits of the meat [17] but also to regulate the chemical composition and sensory properties of Baranjski kulen (PGI) as the final product. These production systems were selected because they represent the actual diversity of pork sources currently used in the production of Baranjski kulen. They differ in both the meat quality traits and in production costs, which affects the economic value and market positioning of the product.

Therefore, the aim of the present study is to evaluate the quality attributes of the traditional meat product Baranjski kulen, focusing on its physicochemical properties, colour characteristics, and textural profile, in relation to the origin of the raw material (i.e., pork) obtained from different production systems.

## 2. Materials and Methods

### 2.1. Animals/Production Chains

The experimental protocol was reviewed and approved by the Bioethics Committee of the Faculty of Agrobiotechnical Sciences Osijek (Approval No. 644-01/23-01/03). All procedures complied with the Croatian Animal Welfare Act and other relevant legal regulations on animal husbandry and welfare.

Raw material for the production of Baranjski kulen came from three pork production chains, which differed in breed and rearing system. In the first pork chain (PC1), pigs of the Black Slavonian pig breed were kept under extensive conditions, fed on pasture to an average live weight of 135 kg, and slaughtered at the age of 18 months. The second production chain (PC2) consisted of crossbred pigs from the Croatian breeding programme (Duroc × Large White), which were kept on straw bedding (deep litter). The fattening pigs in this chain had unlimited access to commercial feed (ad libitum) and were slaughtered at an average weight of 180 kg and an age of 12 months. The last production chain in this study (PC3) was commercial hybrid pigs PIC (Pig Improvement Company; Hendersonville, TN, USA; P337 (Pietrain × Duroc × Pietrain) × Camborough 23), which were kept under industrial conditions up to an average live weight of 160 kg (which they reached in 6 months). From each of the mentioned pork chains, 16 pigs (8 gilts and 8 castrates) were selected as a source of raw material (meat and fat) for the production of Baranjski kulen. At the end of the fattening period, the pigs were transported to a commercial abattoir and slaughtered after stunning with CO_2_. After 24 h of cooling, ham and shoulder were sectioned from the carcasses and used to produce Baranjski kulen (PGI) according to the recipe described in detail in the product specification. The sausages were prepared from pork meat and back fat, with the addition of salt, sweet and hot paprika, garlic, and black pepper, in line with the Baranjski kulen PGI specification. No nitrites were used. The fermentation and ripening process lasted approximately 90–120 days, starting at 18–20 °C and 85–90% relative humidity for the first week, followed by gradual reduction to 12–15 °C and 75–80% humidity until the end of ripening.

### 2.2. Physicochemical Traits of Baranjski Kulen

The weight loss of the sausages was calculated by dividing the difference between their initial weight when fresh and their weight after the ripening period by the initial weight and expressed as percentage.

The pH value was measured in a distilled water homogenate (1:10) of the sample (10 g) using the digital pH metre HI 99613 (Hanna Instruments Inc., Woonsocket, RI, USA). Water activity (aw) was determined using a Rotronic Hygrolab 3 at room temperature (20 ± 2 °C) according to the user manual (Rotronic AG, Bassersdorf, Switzerland).

Chemical composition of sausages was determined using standard analytical methods as follows: ISO 1442:2023 method [18] was used to determine the moisture content, ISO 1443:1973 [19] for the fat content, and ISO 937:2023 [20] for the nitrogen content. To determine the fat content, the Soxhlet method ISO 1443:1973 [19] was used, where samples were digested by acid hydrolysis and the fats extracted with petroleum ether using an automated Soxtherm 2000 device (Gerhardt, Munich, Germany).

The colour parameters (CIE L*, a*, b*, C* and h°) were measured using a Minolta CR-400 colourimeter (Minolta Camera Co., Ltd., Osaka, Japan). Chroma (C*) was calculated as C* = (a^2^ + b^2^)^1/2* and hue angle (h°) as h° = arctan (b*/a*) expressed in degrees, using the arctan2 function to account for the correct quadrant. A white ceramic plate with a D65 light source and a 2-degree standard observer was used for calibration. The colour properties of the sausages were determined using the CIE-Lab colour space, whereby three colour measurements were taken on each cut surface of each sliced sausage.

The texture of the product was measured using a 25 mm diameter cylindrical sample (P/25) connected to the TA.XTplus texture analyser (Stable Micro Systems, Godalming, UK). In total, six samples per sausage were analysed for hardness, elasticity, cohesiveness, gumminess, and chewing resistance using the Texture Exponent 4.0 programme (Stable Microsystems, Godalming, UK).

### 2.3. Statistical Analysis

The GLM procedure in Statistica v.14 software [21] was used to examine the effect of the pork production chain (PC1–PC3) as a fixed factor on the physicochemical traits, colour parameters, and texture profile of Baranjski kulen samples. Pigs of both sexes (gilts and castrates) were included in equal proportion (1:1) within each chain to avoid bias. It should be noted, however, that sex was not included as a factor in the model, since it is not considered relevant for kulen production. Group comparisons were made using Tukey’s range test (*p* < 0.05)

## 3. Results and Discussion

### 3.1. Physicochemical Traits

The physicochemical properties of Baranjski kulen (PGI) from three different pork chains are shown in Table 1.

No differences in weight loss between the samples were observed (*p* > 0.05). The pH values of Baranjski kulen from the different production chains were similar and within the expected range for this type of product. Lower pH values are crucial during fermentation to ensure colour stability, optimal consistency, product stability, taste, and aroma [6,9]. Additionally, as noted by Honikel and Hamm (1994) [22], reduction in the pH to the isoelectric point of actomyosin (around 5.3) is essential for the minimisation of water retention and acceleration of the drying process. Thirumdas et al. (2018) [23] reported higher pH for chorizo sausage, with pH ranging from 5.83 to 5.97. The difference in pH between the results of the present study and the chorizo sausage can be attributed to the different production technology, which mainly refers to the meat and fat content and the differences in the addition of spices to the sausages, as well as the duration of the drying processes. Similarly to the results of this study, Karolyi (2011) [24] reported average pH values of 5.37 for Slavonian kulen samples, a sausage very similar to Baranjski kulen, collected from different locations and of unknown origin.

Baranjski kulen from PC1 meat exhibited a water activity of 0.85, while kulen from the other two production chains (PC2 and PC3) exhibited a slightly lower water activity (0.84); no significant differences between samples were observed for this trait. A low value of water activity inhibits the action of harmful microorganisms, which is crucial for the hygienic conformity of the product itself [25]. According to Heinze and Hautzinger (2007) [26], the water activity in fermented cured meat products should be between 0.70 and 0.91, while Laranjo et al. (2015) [27] found the threshold value of water activity with an inhibitory microbiological effect to be 0.91. Our results show that Baranjski kulen from all three different production chains meets the above criteria and that the risk of growth of pathogenic bacteria that can lead to spoilage of the product is minimised. Pleadin et al. (2021) [28] and Karolyi (2011) [24] reported lower values for water activity in Slavonski kulen, ranging from 0.77 to 0.84 and 0.82, respectively. The authors concluded that the water activity and pH values place kulen in the category of products with low acidity, whose microbiological stability and sustainability is determined by the low water activity in the end-product.

Baranjski kulen from PC1 chain had significantly lower protein content (30.19) compared to PC2 (35.46) and PC3 (35.90) production chains. On the other hand, the highest fat content was found in Baranjski kulen from PC1 (25.97), while sausages from PC2 and PC3 did not differ (*p* > 0.05) in this trait. Pleadin et al. (2014) [29] reported lower percentages of water (29.88%) and fat (23.04%), and a higher percentage of total protein (40.99%) in Slavonski kulen compared to the results of the present study. However, the raw materials (meat and fat) and ripening conditions were significantly different, so these variations are not unexpected. Similarly, Vuković et al. (2012) [30] found lower water (28.2%) and fat (32.6%) contents in Lemeški kulen, which is made from similar ingredients as Baranjski kulen, than those determined in this investigation. It should be noted, however, that their samples came from different producers and lacked strict control over the amount and concentration of ingredients (meat, fat, spices). Additionally, Ikonić et al. (2021) [31] reported higher protein content in Sremski and Lemeški kulen (38.1% and 36.6%, respectively) and lower fat content (14.6% and 21.5%) compared to the results of our study. The observed differences could be attributed to the pig breeds used in sausage production, which were not specified in the study by Ikonić et al. (2021) [31]. On the other hand, Karolyi (2011) [24] reported an average protein content of 35% in Slavonski kulen they investigated, while in investigation of Thirumdas et al. (2018) [23] the average protein content for chorizo sausage was between 30.56% and 35.62%.

Baranjski kulen from the PC1 production chain had the highest (*p* < 0.05) fat content compared to the other two production chains, which did not differ significantly from each other in this trait. The increased fat content can be attributed to intramuscular fat (IMF), a characteristic feature of the Black Slavonian pig breed [32].

### 3.2. Colour

Results of the colour analyses for Baranjski kulen originating from different production chains are presented in Table 2.

The colour of the surface of traditional dried meat products is the result of chemical reactions that occur during smoking and drying of the product [33]. It serves both as an indicator of the success of individual production steps and a key criterion that consumers consider when selecting a particular Baranjski kulen for consumption. It is well-established that moisture loss from the product increases the concentration of pigments such as myoglobin [34], which directly influences the degree of paleness (L*), as observed in meat from various sources [35,36]. In Table 1, it can be observed that Baranjski kulen produced from the meat of PC1 pigs had the lowest moisture content, which could explain the lowest L* values found in the product. Additionally, it is widely accepted that extensive rearing increases the proportion of oxidative fibres in animal muscles, thereby enhancing the red colour of their meat [35]. The longer this rearing practice is maintained, the more pronounced is its effect on muscle fibres in the fresh meat [37]. However, this more pronounced red colour disappears in dry/fermented products due to drying and consequent degradation of myoglobin and other biochemical processes associated with meat processing; this finding was also reported by Denzer et al. (2022) [38]. The lowest values for L* and a* observed sausages made from PC1 support the above hypothesis [39]. In the study on quality attributes of Slavonski kulen, Kovačević et al. (2010) [40] determined that L* in this product ranged from 30.7 to 42.42, which is lower than in the products of a similar type. They explained this by longer ripening period, characteristic for Slavonian kulen. Furthermore, the authors found that the values of a* ranged from 15.15 to 23.34 and b* from 13.57 to 26.4, which is higher than the values observed in present study. However, it is important to note that the genetic background of the pigs and the specifics of their rearing were not specified in their study, so comparisons with the results of the present study should be made with caution. According to Petrović et al. (2007) [41], the L* value of a product similar to the Slavonian and Baranjski kulen, the Petrovská klobása, is expected to be between 32 and 37. Vuković et al. (2012) [30] reported L* values between 31.86 and 33.74, a* values between 19.39 and 25.80, and b* values between 15.52 and 21.17 in Lemeški kulen, which are lower than the results of the current study. Similarly, Ikonić et al. (2021) [31] found lower L* values in Sremski and Lemeški kulen compared to our results, with values of 33.7 and 31.0, respectively.

It can be seen from Table 2 that Baranjski kulen from PC1 had lower (*p* < 0.05) values for colour saturation (C*) and hue angle (h°), indicating a higher proportion of metmyoglobin (MMb) and thus a less intense red colour compared to PC2 and PC3. The formation of more MMb and the resulting discoloration of the product could be attributed to the higher proportion of Type I oxidative fibres, which are typically found in the meat of older pig breeds belonging to the fatty or meat-fatty types, such as the Black Slavonian pig breed [6,16].

In the study on Petrovská klobása by Škaljac et al. (2019) [42], the authors reported slightly higher hue angle values compared to all the groups in our study. They found the hue angle to range from 41.08 to 45.55, with its value dependent on the product’s storage duration. Additionally, the authors observed that the chroma value (C*) ranged from 30.70 to 36.12, which is lower than the values found in the present study for the PC2 and PC3 Baranjski kulen.

### 3.3. Textural Properties

The analysis of the texture profile of Baranjski kulen produced from pork originating from three production chains is shown in Table 3.

The lowest value for hardness was found in Baranjski kulen from PC3 pigs, which can thus be considered the product with the softest texture. The difference in softness between the sausages from the three pork chains can be mainly attributed to the differences in the composition of the fat, i.e., their fatty acid profile. In our previous studies, we found that the meat of fattening pigs from the Black Slavonian pig breed had a higher (*p* < 0.05) ratio of polyunsaturated (PUFA) and monounsaturated fatty acids (MUFA) than the meat of PIC (Pig Improvement Company) hybrid pigs [43]. Although PC2 kulen had the highest moisture content, it also contained relatively more protein and less fat, which favoured the development of a denser protein matrix during fermentation and drying. This compact structure was able to retain water while still increasing hardness, cohesiveness, and chewiness, explaining the apparently contradictory results. The most important substrates for oxidation in meat products are unsaturated fatty acids. Although oxidation is a generally undesirable process, a certain degree of oxidation in production of dry-fermented sausages is beneficial as it improves their characteristic flavour and aroma [44,45]. On the other hand, unsaturated fatty acids, including MUFA and PUFA, have a significant impact on water loss during sausage ripening due to their susceptibility to oxidation because it disrupts protein structures, reducing the ability of the sausage matrix to retain water [46]. In addition, the study by Škrlep et al. (2019) [47] found the same trend of decreasing the hardness of traditional products (sausages) with an increase in the proportion of saturated fatty acids.

Cohesiveness in fermented sausages refers to the ability of the sausage matrix to resist deformation and hold together as a uniform structure. It is a measure of the sausage resistance to breaking under mechanical action and reflects the integrity of the protein, fat, and water network in the product. It is influenced by several factors, including pH value, water, and fat content of the sausage [13,14]. Gimeno et al. (2000) [48] found that the parameters of the texture profile, especially cohesiveness, are negatively correlated with the pH of the product, i.e., when the pH drops below the isoelectric point of the meat, a higher proportion of the protein is extracted, which alters the texture of the product, especially its hardness. The results of this study support this statement. Although the differences in pH among the groups were not statistically significant, Baranjski kulen from the PC2 pig chain had the lowest pH values, below the isoelectric point (Table 1). At the same time, it showed the highest hardness and cohesiveness (*p* < 0.05) compared with Baranjski kulen from the other two pork chains. The same observation regarding the change in the parameters of the texture profile in relation to the change in pH in the product was made for dry/fermented products made from the meat of the Cinta Senese breed [49] and for chorizo sausage [50].

Stajić et al. (2017) [51] analysed the texture of Sremska sausage produced from local pig breeds (Moravka and Mangalitsa) and the commercial Landrace breed. Similarly to the present study, their results showed significant differences in the textural properties of the sausages depending on the genotype [52] of the pig used [53]. The highest hardness was observed in sausages made from the meat of the autochthonous Moravka breed. In addition, the authors found that sausages from indigenous pig breeds had significantly lower values for hardness and chewing resistance compared to sausages from commercial pig breeds. In a related study, Kovačević et al. (2010) [40] reported that salt had a significant effect on the textural properties of Slavonski kulen. However, in contrast to the results of this study, no significant effects on fat content or pH were found.

The results of the texture profile analysis (TPA) showed significant differences in the gumminess of the sausages produced from pork from the three production chains. The highest gumminess values were observed in sausages from the PC2 production chain, followed by those from PC1 and PC3 (Table 3).

As in the case of other TPA parameters, gumminess is also connected to protein and moisture content [48]. Dry-fermented sausages with lower fat content and higher moisture tend to be softer, more gel-like texture, while those with low moisture tend to have a crumblier texture [54,55]. The results of the present study support this observation.

In the present study, Baranjski kulen made from the PC2 production system exhibited the highest chewiness values, followed by sausages produced from the PC1 and PC3 production systems (*p* < 0.05). Chewiness, like other textural properties, is closely related to the collagen content of the pork [56]. Sausages with higher protein content have increased chewiness as shown by Ikonić et al. (2020) [31], who found that samples of Lemeški kulen, which had the highest hardness values and the highest protein content, also exhibited the highest chewiness values.

## 4. Conclusions

Comparative analysis of Baranjski kulen (PGI) samples from diverse production chains showed clear differences in their physicochemical properties, colour, and texture. Baranjski kulen produced from the PC2 and PC3 chains had a higher protein content and a lower fat content than the PC1 kulen. In addition, the PC3 kulen exhibited higher colour intensity and differentiation, indicating a more pronounced visual appeal. In terms of texture, Baranjski kulen from PC2 pork chain exhibited the highest hardness, cohesiveness, gumminess, and chewiness, indicating a denser and more challenging bite. In contrast, Baranjski kulen from the PC3 pork chain exhibited a softer, more delicate texture. Baranjski kulen from the PC1 production chain displayed intermediate values for all textural attributes examined.

The results of the study demonstrate that different pork production chains lead to the production of Baranjski kulen with distinct physicochemical, colour, and textural properties. Rather than indicating that strict quality control could lead to uniformity, the findings highlight that recognised production chains can be used to produce sausages with specific and diverse quality profiles. Such diversity enriches the range of traditional PGI products available to consumers, who are known to value authenticity and heritage over convenience or standardisation. Therefore, the study underlines the role of production chain choice in shaping the final product characteristics, rather than providing evidence for process optimisation or strategies to enhance market value.

## Figures and Tables

**Table 1:** Means and standard deviations (in brackets) for physicochemical traits of Baranjski kulen (PGI) from three different pork chains.

Parameter	Production Chain		
	PC1	PC2	PC3
Weight loss, %	51.8 (5.48)	47.62 (3.45)	45.79 (1.67)
pH	5.33 (0.24)	5.26 (0.04)	5.30 (0.08)
Water activity (a_w_)	0.85 (0.05)	0.84 (0.04)	0.84 (0.04)
Moisture, %	35.49 (4.48)	38.22 (2.35)	36.80 (1.60)
Protein, %	30.19 ^a^ (3.69)	35.46 ^b^ (1.76)	35.90 ^b^ (1.27)
Fat, %	25.97 ^b^ (4.43)	18.29 ^a^ (1.08)	19.00 ^a^ (2.04)

PC—pork production chain; ^a,b^ letters with different superscripts differ (*p* < 0.05).

**Table 2:** Means and standard deviations (in brackets) for colour parameters of Baranjski kulen (PGI) from three pork chains.

Parameter	Production Chain		
	PC1	PC2	PC3
L*	37.13 ^c^ (1.58)	40.24 ^b^ (3.73)	42.90 ^a^ (0.97)
a*	17.21 ^c^ (1.55)	19.78 ^b^ (3.36)	22.39 ^a^ (0.47)
b*	13.85 ^c^ (1.62)	19.19 ^b^ (5.68)	23.48 ^a^ (1.05)
C*	22.09 ^c^ (2.19)	27.62 ^b^ (6.32)	32.44 ^a^ (1.05)
h°	38.76 ^c^ (1.28)	43.28 ^b^ (4.19)	46.35 ^a^ (0.81)

PC—pork production chains; L*—lightness; a*—redness; b*—yellowness; C*—chroma (colour saturation); h°—hue angle; ^a,b,c^ letters with different superscripts differ (*p* < 0.05).

**Table 3:** Means and standard deviations (in brackets) for texture profile of Baranjski kulen (PGI) made from pork originating from three pork chains.

Parameter	Production Chain		
	PC1	PC2	PC3
Hardness, N	131.75 ^b^ (2.753)	200.12^c^ (2.37)	73.38 ^a^ (7.12)
Elasticity	0.67 (0.05)	0.65 (0.04)	0.63 (0.03)
Cohesiveness, N	0.32 ^a^ (0.02)	0.38 ^b^ (0.02)	0.30 ^a^ (0.03)
Gumminess	42.42 ^b^ (10.15)	81.66 ^c^ (9.22)	22.67 ^a^ (3.13)
Chewiness	2873.96 ^b^ (6.47)	5266.52 ^c^ (5.37)	1447.67 ^a^ (0.22)

PC—pork production chains; ^a,b,c^ letters with different superscripts differ significantly (*p* < 0.05).

## Data Availability

The data presented in this study are available on request from the corresponding author. The data are not publicly available due to the restrictions imposed by the funding agencies.
